# Weisheng-tang protects against ischemic brain injury by modulating microglia activation through the P2Y12 receptor

**DOI:** 10.3389/fphar.2024.1347622

**Published:** 2024-09-04

**Authors:** Min Jae Kim, Dohee Lee, Ji Hye Ryu, Seo-Yeon Lee, Byung Tae Choi, Young Ju Yun, Hwa Kyoung Shin

**Affiliations:** ^1^ Department of Korean Medical Science, School of Korean Medicine, Pusan National University, Yangsan, Gyeongnam, Republic of Korea; ^2^ Graduate Training Program of Korean Medical Therapeutics for Healthy-Aging, Pusan National University, Yangsan, Gyeongnam, Republic of Korea; ^3^ Department of Pharmacology, Wonkwang University School of Medicine, Iksan, Jeonbuk, Republic of Korea; ^4^ Department of Korean Medicine, School of Korean Medicine, Pusan National University, Yangsan, Gyeongnam, Republic of Korea

**Keywords:** middle cerebral artery occlusion, ischemia, microglia activation, P2Y12 receptor, Weisheng-tang

## Abstract

**Background:** Stroke, a leading cause of death and disability, lacks effective treatments. Post-stroke secondary damage worsens the brain microenvironment, further exacerbating brain injury. Microglia’s role in responding to stroke-induced damage in peri-infarct regions is crucial. In this study, we explored Weisheng-tang’s potential to enhance ischemic outcomes by targeting microglia.

**Methods:** We induced middle cerebral artery occlusion and reperfusion in mice, followed by behavioral assessments and infarct volume analyses after 48 h, and examined the changes in microglial morphology through skeleton analysis.

**Results:** Weisheng-tang (300 mg/kg) significantly reduced infarction volume and alleviated neurological and motor deficits. The number of activated microglia was markedly increased within the peri-infarct territory, which was significantly reversed by Weisheng-tang. Microglial morphology analysis revealed that microglial processes were retracted owing to ischemic damage but were restored in Weisheng-tang-treated mice. This restoration was accompanied by the expression of the purinergic P2Y12 receptor (P2Y12R), a key regulator of microglial process extension. Weisheng-tang increased neuronal Kv2.1 clusters while suppressing juxtaneuronal microglial activation. The P2Y12R inhibitor—ticagrelor—eliminated the tissue and functional recovery that had been observed with Weisheng-tang after ischemic damage.

**Discussion:** Weisheng-tang improved experimental stroke outcomes by modulating microglial morphology through P2Y12R, shedding light on its neuroprotective potential in ischemic stroke.

## 1 Introduction

Stroke, a leading cause of mortality and long-term disability (Lackland et al., 2014), is a major global health concern. It primarily results from sudden interruption of the blood supply to the brain, which can lead to ischemic damage and a cascade of pathophysiological events (Dirnagl et al., 1999). Currently, therapeutic options for stroke are limited, necessitating the development of novel and effective drug treatments to ameliorate stroke symptoms and their long-term consequences. Neuroinflammation is central in resolving secondary injuries after ischemic brain insult (Kang et al., 2024). Consequently, regulating the inflammatory responses is a promising therapeutic approach for stroke. To this end, preventing additional neuroinflammation-induced brain damage is a crucial stroke treatment strategy.

Recent research has revealed the pivotal role of microglia, which are resident immune cells that maintain homeostasis, regulate neuronal survival, and modulate synaptic plasticity, in healthy and diseased brain states (Guilarte et al., 2016; Thion et al., 2018). Microglia’s responsiveness to ischemic brain injury is well documented, and the timing of ischemic damage and the microglial activation state determine the harmful role of microglia in ischemic brain (Gaire, 2022). They orchestrate subsequent inflammatory processes and contribute to tissue damage (Wang et al., 2007; Ceulemans et al., 2010). However, microglia can also play a crucial neuroprotective role via the production of anti-inflammatory cytokine and growth factors (Bodhankar et al., 2013; Serhan et al., 2019). Activated microglia exhibit distinct morphological features, including an enlarged Soma or retracted process as they migrate to the injury sites, release inflammatory cytokines, and engage in the phagocytosis of injured neurons (Fu et al., 2014). Considering these insights, targeting microglia and modulating their functions have emerged as promising avenues for developing new stroke treatments.

In the ischemic brain, damaged neuron cells release adenosine 5′-triphosphate (ATP) or adenosine 5′-diphosphate (ADP), and microglial processes extend toward the ATP released from the injured neurons through the metabotropic ATP receptor P2Y12R (Ohsawa et al., 2010). P2Y12R was initially known for mediating platelet aggregation; however, studies have demonstrated the relationship between platelet function and microglial morphology (Andre et al., 2003). Increased neuronal activation results in microglial processes covering neuronal cell bodies, dependent on P2Y12R expression (Cserép et al., 2020). Furthermore, P2Y12R was proposed as a potential marker of anti-inflammatory microglial activation in ischemic rodents and human brains (Villa et al., 2018). Consequently, P2Y12R may be involved in regulating microglial morphological changes and neuronal activity. Developing pharmaceutical interventions targeting this pathway could be a viable stroke treatment strategy.

Weisheng-tang, a traditional prescription, has been used to treat dyspepsia and fatigue, as documented in *Dongui Bogam* (Heo, 2013). Our previous study demonstrated its potential as a neuroprotective agent (Kim et al., 2019). We also previously uncovered Weisheng-tang’s significant capacity to reduce ischemic damage by protecting the blood–brain barrier in a murine model of permanent ischemic injury, highlighting its potential as a therapeutic intervention. Thus, in this study, we investigated Weisheng-tang’s effects on cerebral ischemia and reperfusion injury. Focusing on microglial responses and potential modulatory effects, we aimed to unravel the mechanisms underlying the neuroprotective properties of Weisheng-tang and assess its potential as a novel approach to stroke management.

We primarily aimed to investigate Weisheng-tang’s ability to regulate stroke-induced microglial modulation in the brain, promoting functional recovery in the middle cerebral artery occlusion and reperfusion (MCAO/R) mouse model. Additionally, we tested the hypothesis that Weisheng-tang promotes microglial morphological changes and activation by regulating the expression level of P2Y12R.Article types.

## 2 Materials and methods

### 2.1 Preparation of Weisheng-tang

Weisheng-tang consists of four botanical drugs as follows; 8g *Astragalus mongholicus* Bunge [Fabaceae; Astragali radix], 4g *Angelica gigas* Nakai [Apiaceae; Angelica gigas radix], *Paeonia lactiflora* Pall [Paeoniaceae; Paeoniae radix alba], 4g *Glycyrrhiza glabra* Linn [Fabaceae; Glycyrrhizae radix et rhizoma]. These botanical drugs were obtained from the Korean Medicine Hospital (Pusan National University) and authenticated by professor Dr. Young Ju Yun (Department of Integrative Medicine, School of Korean Medicine, Pusan National University). Weisheng-tang (total 61.87 g) was boiled twice in 1.2 L of distilled water at 120°C ± 5°C for 3 h, passed twice through filter paper (Advantech, Tokyo, Japan), and concentrated using an evaporator equipped with a decompression device (EYELA Co., Tokyo, Japan). The samples were freeze-dried (Labconco, Kansas City, MO, United States), and 13.8 g was obtained in powder form. For administration, Weisheng-tang was dissolved in phosphate-buffered saline (PBS).

### 2.2 Animal experiment

Six-week-old male C57BL/6 mice were obtained from Hana Biotech (Ansan, South Korea). The mice were housed under a 12-h light/dark cycle and provided *ad libitum* access to food and water. The Pusan National University Institutional Animal Care and Use Committee (PNU-IACUC) reviewed the animal protocols in this study and validated their findings (PNU-2020-2524 and PNU-2020-2826). A total of 120 mice (16 control and 104 with MCAO/R) were included and randomly divided into different experimental groups. The minimized animal sample size was calculated using G*Power 3.1 software (Heinrich-Heine-Universität, Düsseldorf, Germany, http://www.gpower.hhu.de/). The sample size for each group was determined based on neurological severity scores from our pilot study results (4 groups each n = 6, effect size f = 0.852, α = 0.05, and β = 0.8). Consequently, for behavioral testing, the sample size for each group was set to a minimum of five animals. All procedures were performed under sterile conditions using aseptic techniques, including the use of sterile gloves and instruments. Mice were randomly divided into different groups and allocated in a blinded manner. For the treatments, mice orally received 0.15 mL of Weisheng-tang extract in 1×PBS at the appropriate concentration once a day for 4 days and 1 h before focal cerebral ischemia. The vehicle group received 1×PBS instead of the Weisheng-tang during the same period. The experimental drug, ticagrelor (3 mg/kg dissolved in 1% carboxymethyl cellulose, Sigma-Aldrich, St Louis, MO, United States) was orally administered for 10 min, 16 h, and 24 h following ischemia.

### 2.3 Cerebral ischemia/reperfusion

MCAO/R causes cerebral ischemia/reperfusion (Kim et al., 2020). The mice underwent deep anesthesia, confirmed by the absence of cardiovascular responses to a tail pinch, using facemask-delivered isoflurane (2% for induction and 1.5% for maintenance) in 80% N_2_O and 20% O_2_. The head and middle neck were shaved, and the exposed skin was disinfected with 70% ethanol and iodine solution (100 mg/mL) before making the incision. A fiber-optic probe was attached to the exposed skull above the left MCA throughout the procedure and during reperfusion, allowing continuous monitoring of regional cerebral blood flow using the PeriFlux Laser Doppler System 5000 (Perimed, Stockholm, Sweden). A 7-0 silicon-coated monofilament (Duccol Corporation, Redlands, CA, United States) was inserted into the internal carotid artery to obstruct the MCA and induce MCAO. A laser Doppler flowmeter (Perimed) was used to confirm that the filament was removed after 60 min to allow for reperfusion. The mice’s body temperature was maintained at 37.5°C throughout the procedure with a Panlab thermostatic heating blanket (Harvard Apparatus, Holliston, MA, United States). Control surgery involved the same procedure without monofilament insertion. Mice were monitored throughout recovery from anesthesia in a heated 30°C cage using a heat lamp regulated by a temperature controller. Mashed chow was placed in a petri dish on the floor of the cage to encourage eating, along with clean bedding. The mice were housed one per cage during the recovery period. Mice were included in the study if they demonstrated successful MCAO/R modeling, defined by a reduction in focal cerebral blood flow to less than 25% of baseline. Mice were excluded from the study due to either death after ischemia or failure to induce ischemia successfully. Nine mice were excluded from further assessments due to death following ischemia, while no animals died from the control procedure. All mice died within 48 h after MCAO/R and before behavior tests were performed. Specifically, the details are as follows: three mice from the vehicle group, one mouse from the 30 mg/kg Weisheng-tang group, two mice from the vehicle group in the ticagrelor experiment, one mouse from the 300 mg/kg Weisheng-tang group, and two mice from the ticagrelor group.

### 2.4 Infarct volume and edema

Mice brains were procured 48 h after MCAO/R. Infarct size was ascertained by staining 2 mm-thick brain sections with 2,3,5-triphenyltetrazolium chloride (TTC). The i-Solution software (Image and Microscope Technology, Vancouver, Canada) was used to quantify the infarct magnitude. The infarct volume was determined by summing the infarcted areas (white) across the five brain sections and multiplying by the section thickness (2 mm), encompassing the portion of the ipsilateral side that had experienced direct injury. Cerebral edema was assessed by subtracting the indirect infarct volume from the infarct volume. The indirect infarct volume was determined using the following formula: contralateral hemisphere volume (mm³) - intact ipsilateral hemisphere volume (mm³).

### 2.5 Behavioral test

The behavioral tests were conducted in a quiet room with a light intensity of less than 50 lux and a temperature of 22°C ± 1°C. All animals were acclimated in the test rooms for over 30 min before testing. All behavioral tests were performed by blinded observers and independent researchers.

#### 2.5.1 Neurological severity scores

The following criteria were used to score the modified neurological severity score (mNSS) 48 h after MCAO/R: one point each for forelimb flexion, hindlimb flexion, and head movement of >10° to the vertical axis in <30 s when the mice were lifted by the tail. If exposed to all contents, the mice could receive up to three points. The mice were then placed on the floor, and their responses were recorded as follows: 0 for regular work, one for failure to walk straight, two for circling the paretic side, and three for falling to the paretic side. The aggregate of the scores from the two portions mentioned above determined the final score, with a limit of six points (Chen et al., 2001).

#### 2.5.2 Wire-grip test

The wire-grip test was performed 48 h after ischemic injury to evaluate vestibular motor function. Each mouse was suspended using its forepaws while dangling on a metal wire. The wire-grip scores were as follows: 1 = not gripping the wire; 2 = utilizing both forepaws and hind paws to grip the wire without using the tail; 3 = gripping without moving using fore and hind paws and the tail; 4 = using both forepaws, both hind paws, and the tail to move on the wire; and 5 = moving smoothly on the wire (Kim et al., 2019).

#### 2.5.3 Rotarod test

The rotarod test (Panlab S.L.U., Barcelona, Spain) evaluated the locomotor function based on the average latency until the mice fell off the spinning rod. Before the actual test, each mouse underwent an adaptation period on the rotating rod for 2 days. During this time, they were pre-trained five times daily, starting at 4 rpm and gradually accelerating to 40 rpm. On the following day, under conditions similar to the actual test, mice were observed at a constant speed of 18 rpm for up to 3 min, with five trials per mouse. The average time from these trials was used to group the mice, ensuring no significant differences between groups. The actual test was then conducted in the same manner as the grouping phase.

### 2.6 Immunofluorescence staining

After 48 h of focal cerebral ischemia, mice were perfused with cold PBS followed by then 4% paraformaldehyde. Their brains were subsequently removed and preserved in 4% paraformaldehyde for 24 h and cryopreserved in 30% sucrose for 72 h at 4°C. Before analysis, the brains were preserved at −80°C in an optical cutting temperature compound (Sakura Finetek, Torrance, California). Sections of the frozen brains (20-μm-thick) were cut with a CM 3050 cryostat (Leica Microsystems, Wetzlar, Germany). The brain sections were immunostained with anti-Iba-1 (1:100, Wako, 019-19741, Osaka, Japan; 1:100, Novus, NB100-1028), P2Y12R (1:200, AnaSpec, AS-55043A, Fremont, CA, United States), 2ʹ,3ʹ-cyclic nucleotide 3ʹ-phosphodiesterase (1:100, Abcam, ab6319, Cambridge, United Kingdom), glial fibrillary acidic protein (1:200, Millipore, MAB360, Billerica, MA, United States), NeuN (1:100, Millipore, MAB377), CD68 (1:100, Bio-Rad Laboratories Inc., MCA1957GA, Hercules, California), and Kv2.1 (1:100, UC Davis/NIH NeuroMab Facility, 75-014, Davis, California) overnight at 4°C. They were subsequently incubated with Alexa 488- (1:500, A-11001, A-11008, A-11055; Life Technologies, Carlsbad, CA, United States), Alexa 594- (1:500, A-11005, A-11037; Life Technologies), or Alexa 647- (1:500, A-31571; Life Technologies) conjugated secondary antibodies for 2 h in the dark. We used 4′, 6-diamidino-2-phenylindole (DAPI, Molecular probes) to stain the nuclei. Laser scanning confocal equipment such as K-1 Fluo (Nanoscope Systems, Daejeon, Korea) and LSM 900 (Carl Zeiss, Oberkochen, Germany) were used to visualize the fluorescence images. ImageJ (Fiji, NIH, Bethesda, MD, United States) and i-Solution (Image and Microscope Technology) were used to quantify the images.

### 2.7 Quantification of confocal images

We employed skeleton analysis of confocal images to analyze the morphological alterations in microglia from fixed brain tissues. Images were captured at 40× magnification using a z-stack and saved at their highest intensities. We first thresholded and individually skeletonized the images of each microglia cell using ImageJ’s “skeletonize” function. Quantitative analysis determined the overall process length, points, and number of branches. We assessed the phagocytic microglia by calculating the percentage of CD68^+^/Iba-1^+^ cells relative to the total Iba-1 density. We used 60× magnification with a z-stack to obtain confocal images and evaluate the microglial coverage and Kv2.1 clusters. Microglial coverage was quantified as the percentage of microglial cells in contact with neuronal cell bodies relative to the total surface area of the neuronal cell bodies. We measured the Kv2.1 cluster’s intensity at sites where microglial processes and neuronal Kv2.1 were identified in individual cells using ImageJ for Kv2.1 cluster analysis.

### 2.8 Western blotting

Protein was isolated from brain tissues using standard techniques and then lysed with radioimmunoprecipitation assay (RIPA) buffer (Cell Signaling, Beverly, MA, United States) supplemented with a protease inhibitor mixture (Genedepot, Katy, TX, United States) and a phosphatase inhibitor mixture (Genedepot). Equal amounts of total protein (50 μg) were separated using 12% sodium dodecyl sulfate-polyacrylamide gel electrophoresis (SDS-PAGE), transferred onto a nitrocellulose membrane (Amersham, Little Chalfort, United Kingdom), and immunoblotted with antibodies specific to P2Y12R (1:1,000, Alomone Labs, APR-012, Jerusalem, Israel) overnight at 4°C. β-Actin (1:4,000, A5316, Sigma-Aldrich, St Louis, MO, United States) was used to confirm equal protein loading. Horseradish peroxidase-conjugated goat anti-rabbit (1:4,000, 4,050–05, Southern Biotech, Birmingham, AL, United States) and anti-mouse IgG (1:4,000, 1,031–05, Southern Biotech) were used as secondary antibodies. The immunoblots were incubated for 1 h. The intensity of chemiluminescence was measured using an ImageQuant LAS 4000 apparatus (GE Healthcare Life Sciences, Uppsala, Sweden). Band intensity was quantified using ImageJ software.

### 2.9 Statistical analyses

Data are presented as the mean ± standard error of the mean (SEM), and all statistical analyses were performed using SigmaPlot 11.2 (Systat Software Inc., San Jose, CA, United States). One-way analysis of variance (ANOVA) followed by the Tukey’s *post hoc* test was performed to distinguish various groups. Statistical significance was set at *p* < 0.05.

## 3 Results

### 3.1 Weisheng-tang protected against brain injury 48 h after MCAO/R

**FIGURE 1:**
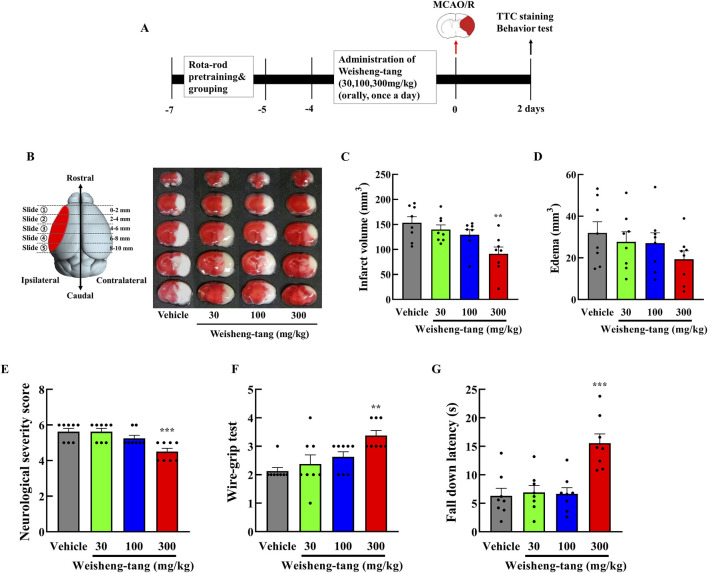
Neuroprotective effects of Weisheng-tang on ischemic stroke. **(A)** Experimental timeline of MCAO/R, Weisheng-tang treatment, and behavioral tests. Mice were orally administered Weisheng-tang (30, 100, and 300 mg/kg) once daily for 4 days and 1 h before MCAO/R, followed by the assessment of behavioral tests and measurement of the infarct volume 48 h after MCAO/R. **(B)** Representative images of brain slices sectioned coronally at 2 mm intervals and stained with 2% TTC, along with quantified analysis of **(C)** the infarct vloume and **(D)** edema. **(E)** Neurological severity score, **(F)** wire-grip test, and **(G)** rotarod test were performed to evaluate functional outcomes. N = 8 each. All data are presented as means ± SEM. Statistical significance was assessed using one-way ANOVA with the Tukey’s *post hoc* test. **p < 0.01, and ***p < 0.001 vs. the vehicle group. Abbreviations: 2,3,5-triphenyltetrazolium chloride (TTC), middle cerebral artery occlusion and reperfusion (MCAO/R).

Mice were orally administered Weisheng-tang (30, 100, or 300 mg/kg) once daily for 4 days and 1 h before MCAO/R. Neurobehavioral assessment and infarct volume measurement were performed 48 h after MCAO/R (Figure 1A). Coronal brain slices, sectioned at 2 mm intervals, were subjected to TTC staining. A dose-dependent reduction in infarct volume was observed, with the most significant reduction occurring in mice pretreated with 300 mg/kg Weisheng-tang (Figures 1B, C, p = 0.003). However, while there was a tendency for edema to decrease at the 300 mg/kg dose, the reduction was not statistically significant (Figure 1D). The Weisheng-tang group exhibited improved neurological outcomes than did the MCAO/R group, as indicated by the mNSS test (Figure 1E). Similarly, the Weisheng-tang-treated group demonstrated enhanced vestibular motor function in the wire-grip test and improved motor coordination in the rotarod test (Figures 1F, G). Overall, the data from TTC staining and neurobehavioral assessments suggest that pretreatment with 300 mg/kg Weisheng-tang enhanced tissue and functional recovery 48 h after MCAO/R. In subsequent experiments, 300 mg/kg of Weisheng-tang was selected for further investigation. To determine whether the preventive effect of Weisheng-tang was long-lasting, we used the same drug administration protocol as in the acute effect experiment. Mice were orally administered Weisheng-tang (300 mg/kg) once daily for 4 days and 1 h before MCAO/R. Long-term neurological and motor outcomes were evaluated 1 and 2 weeks post-stroke (Supplementary Figure S1). We did not observe any long-term therapeutic effect of Weisheng-tang in neurological and motor function tests. Collectively, these findings indicate that Weisheng-tang pretreatment markedly attenuated the neurological and motor deficits 48 h after focal cerebral ischemia, but its preventive effect does not appear to be long-lasting.

### 3.2 Weisheng-tang reduced phagocytotic microglia count and modulated microglia morphology in the ischemic penumbra

**FIGURE 2:**
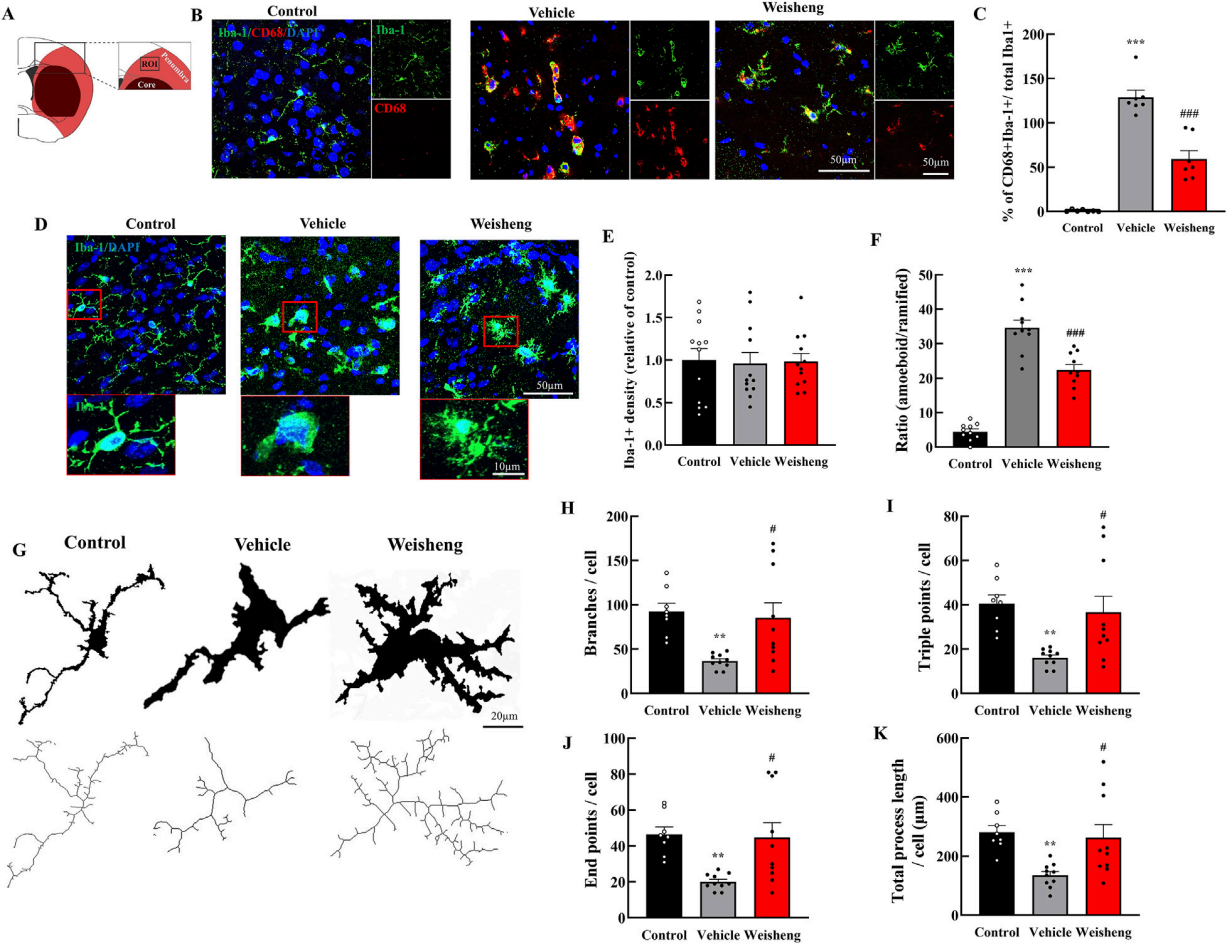
Weisheng-tang reduced phagocytic microglia and modulated microglial morphology in the penumbra region. **(A)** Illustration of the ROI for observing microglia in the ischemic brain. **(B)** Representative photographs of phagocytic microglia stained for CD68 and Iba-1 in ROI 48 h after MCAO/R. **(C)** Quantification of phagocytic microglia (CD68^+^/Iba-1^+^ cells/total microglia, N = 7 images each). **(D)** Representative images of microglia visualized via Iba-1 staining in the ischemic brain, and the red boxes highlight the microglial morphology of the three groups. **(E)** Microglia cell density quantification in the penumbra region (N = 12 images each). **(F)** The ratio of amoeboid and ramified microglia was morphologically analyzed (N = 10 images each) **(G)** Each microglia cell from confocal images was altered with skeletonization using the “skeletonize” plugin by ImageJ to measure their morphology. Quantification for **(H)** branches, **(I)** triple points, **(J)** endpoints, and **(K)** total process length (N = 8–10 each). All data are presented as means ± SEM. Statistical significance was assessed using one-way ANOVA with the Tukey’s *post hoc* test. **p < 0.01, ***p < 0.001 vs. the control group. #*p* < 0.05, ###*p* < 0.001 vs. the vehicle group. Abbreviations: Region of interest (ROI), 4′,6-diamidino-2-phenylindole (DAPI), ionized calcium-binding adaptor molecule-1 (Iba-1), cluster of differentiation 68 (CD68), and 300 mg/kg Weisheng-tang (Weisheng).

We initially assessed the quantity and phenotype of microglia in the ischemic penumbra to investigate Weisheng-tang’s potential effect on microglia in the brain following MCAO/R (Figure 2A). The number of phagocytic microglia—CD68^+^/Iba-1^+^ cells— was higher in the MCAO/R group than in the control group. Weisheng-tang significantly reduced the number of CD68^+^/Iba-1^+^ cells induced by MCAO/R (Figures 2B, C). No difference existed in Iba-1 expression; nonetheless, we observed morphological changes in the microglia among the three groups (Figures 2D, E). Additionally, we quantified the ratio of amoeboid microglia, characterized by a large round Soma and short branches, to ramified microglia, which have a small Soma and long processes. Morphological analysis revealed a significant decrease in the ratio of amoeboid microglia in the Weisheng group compared to the vehicle group (Figure 2F). We quantified the number of branches, endpoints, triple points, and total process length using skeleton analysis in ImageJ with 400× magnification confocal images to evaluate these morphological alterations (Figure 2G). The MCAO/R-induced microglial activation, typically characterized by a transition from a highly ramified to an amoeboid morphology, was significantly reversed in the Weisheng-tang group (Figures 2H–K). These findings suggest that Weisheng-tang facilitated the restoration of activated microglia from the amoeboid form induced by ischemic stroke through morphological changes.

### 3.3 Weisheng-tang primarily increased P2Y12R expression on the microglia

**FIGURE 3:**
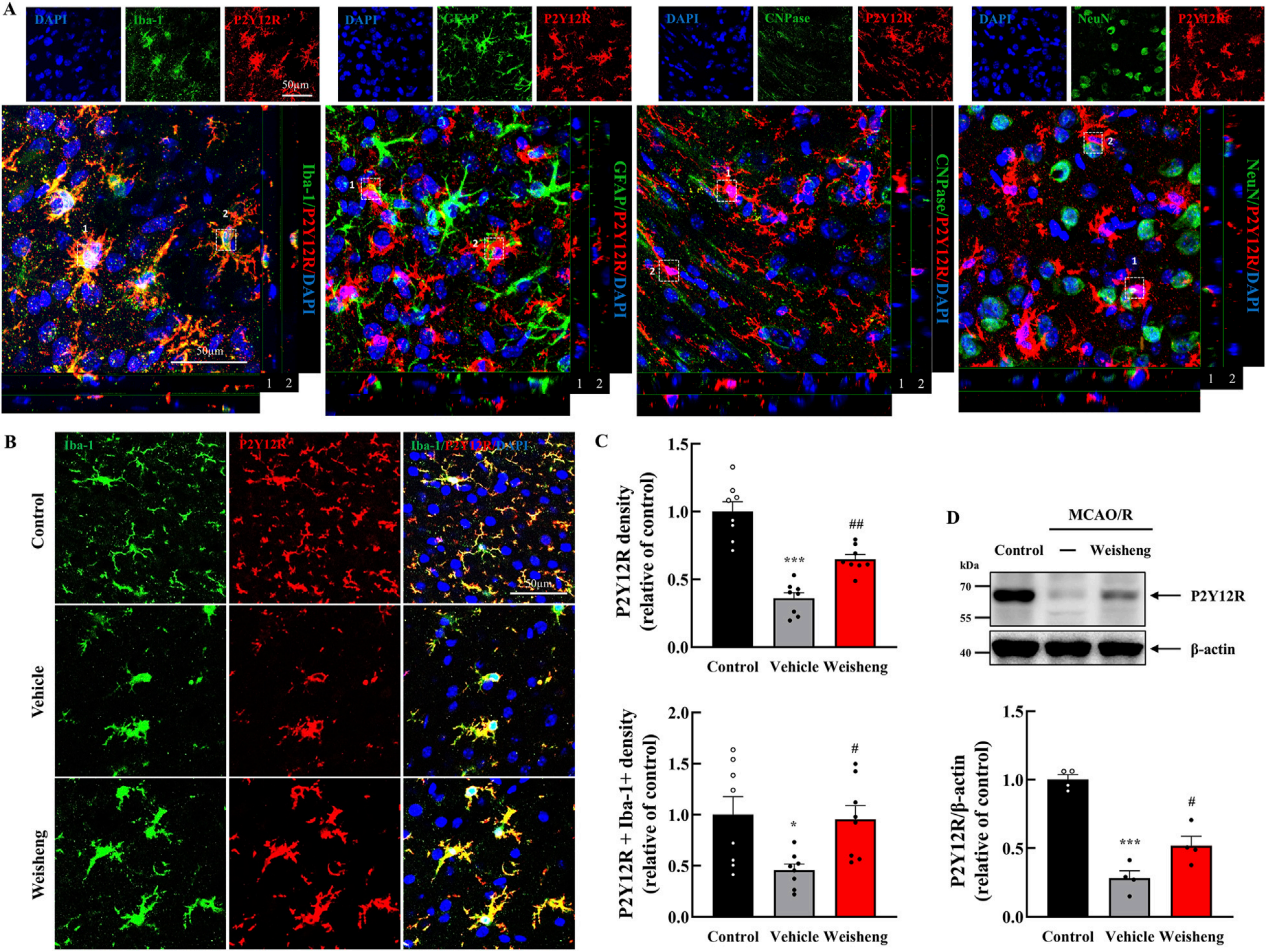
P2Y12R immunoreactivity colocalized with upregulated microglia process via Weisheng-tang. **(A)** Representative confocal images displaying P2Y12R expression along with various markers such as Iba-1, GFAP, CNPase, and NeuN to verify the cell types in which P2Y12R was expressed. White boxes indicate whether P2Y12R is expressed in each cell. **(B)** Representative images of P2Y12R-expressing microglia in the ischemic brain. **(C)** Quantification of P2Y12R^+^ or P2Y12R^+^/Iba1^+^ cell density 48 h after MCAO/R (N = 8 images each). **(D)** Representative immunoblots of P2Y12 protein expression. The band intensities are presented relative to those of β-actin (N = 4 each). All data are presented as means ± SEM. Statistical significance was assessed using one-way ANOVA with the Tukey’s *post hoc* test. *p < 0.05, ***p < 0.001 vs. the control group, #*p* < 0.05, ##*p* < 0.01 vs. the vehicle group. Abbreviations: 4′,6-diamidino-2-phenylindole (DAPI), ionized calcium-binding adaptor molecule-1 (Iba-1), glial fibrillary acidic protein (GFAP), 2ʹ,3ʹ-cyclic-nucleotide 3ʹ-phosphodiesterase (CNPase), neuronal nuclear protein (NeuN), and P2Y12 receptor (P2Y12R), and middle cerebral artery occlusion and reperfusion (MCAO/R), and 300 mg/kg Weisheng-tang (Weisheng).

We explored whether purinergic P2Y12R plays a role in microglial morphological changes induced by Weisheng-tang to elucidate how it facilitates microglial process extension in the ischemic area. Initially, we examined whether P2Y12R immunoreactivity was colocalized with Iba-1 (a microglia marker), GFAP (an astrocyte marker), CNPase (an oligodendrocyte marker), and NeuN (a neuron cell marker). We observed that P2Y12R was primarily expressed in the microglia (Figure 3A). Furthermore, P2Y12R^+^ and P2Y12R^+^/Iba-1^+^ cells were downregulated in the peri-infarct area after MCAO/R compared to that in the control group with a significantly reversed downregulation in the Weisheng-tang group (Figures 3B, C). Similarly, in the Western blot analysis, the P2Y12R protein increased in the Weisheng-tang group compared to the vehicle group (Figure 3D). These findings suggest that Weisheng-tang promoted the restoration of retracted microglial processes in association with P2Y12R expression, a crucial regulator of microglial process extension.

### 3.4 Weisheng-tang reduced microglia-neuron interaction

**FIGURE 4:**
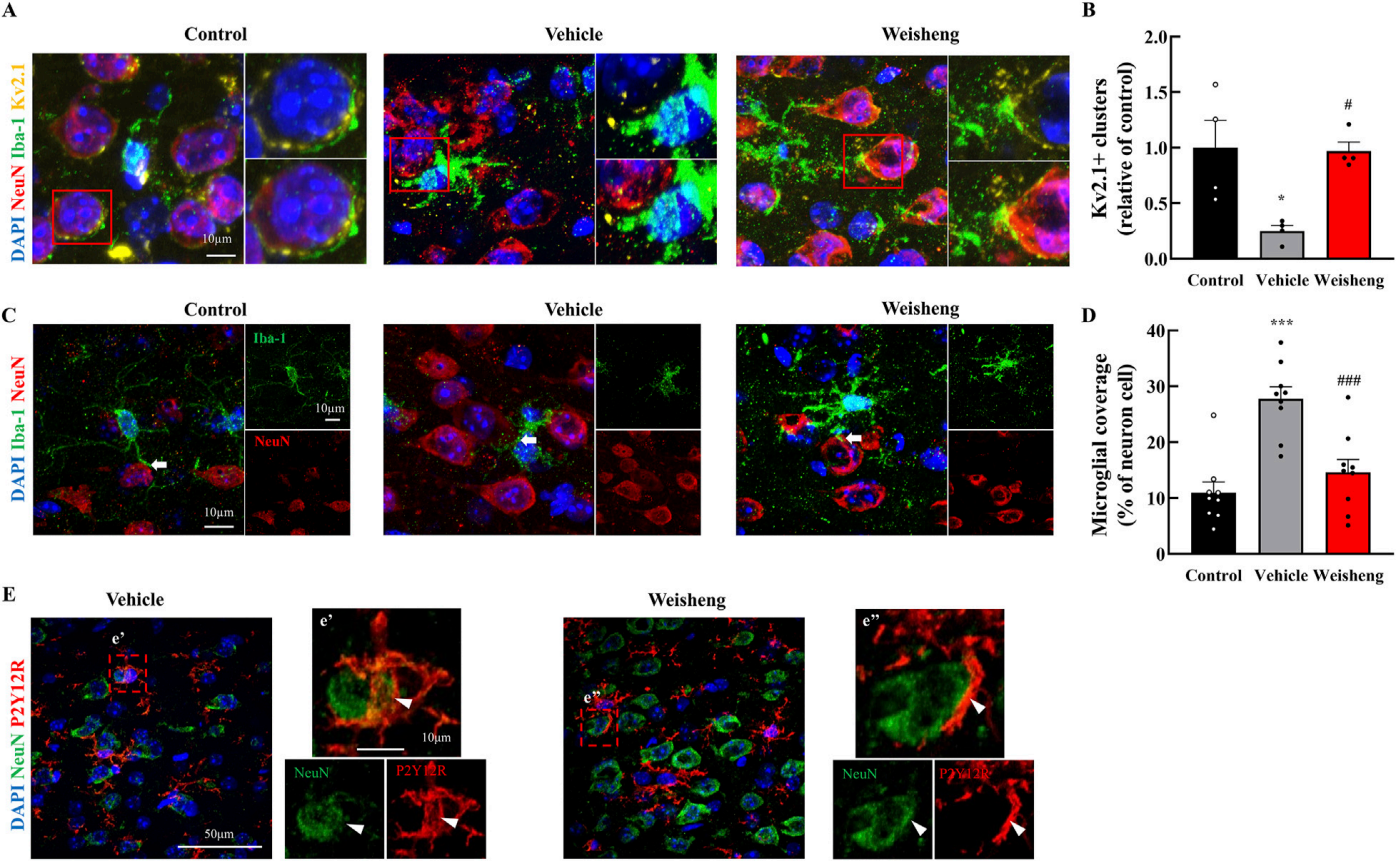
Weisheng-tang regulated Kv2.1 clusters and microglial coverage 48 h after MCAO/R. **(A)** Representative triple fluorescent images for Iba-1, NeuN, and Kv2.1 in the penumbra region of the ischemic brain. **(B)** Quantification of Kv2.1 clusters in the penumbra region 48 h after MCAO/R (N = 4 each). **(C)** Representative images displaying microglial coverage of neuronal cells, with arrows indicating microglial processes in physiological contact with neurons. **(D)** Quantification of the percentage of microglial coverage of neuronal cells (N = 9 images each). **(E)** Representative images presenting P2Y12R coverage with neuronal cells (indicated by the arrowhead) in the Weisheng group. All data are expressed as means ± SEM. Statistical significance was assessed using one-way ANOVA with the Tukey’s *post hoc* test. *p < 0.05, ***p < 0.01 vs. the control group, #*p* < 0.05, ###*p* < 0.001 vs. the vehicle group. Abbreviations: 4′,6-diamidino-2-phenylindole (DAPI), ionized calcium-binding adaptor molecule-1 (Iba-1), P2Y12 receptor (P2Y12R), neuronal nuclear protein (NeuN), and voltage-gated potassium (Kv), and 300 mg/kg Weisheng-tang (Weisheng).

We aimed to determine whether the increase in microglial processes induced by Weisheng-tang protected neuronal function following ischemic injury. First, we measured neuronal density using NeuN staining and observed a significant increase in NeuN^+^ cells in the Weisheng-tang-treated group, which were reduced by MCAO/R (Supplementary Figure S3). Next, we explored how Weisheng-tang influences the interaction between microglia and neurons, focusing on clustering voltage-gated potassium (Kv) 2.1 and microglial coverage around neurons. Kv2.1^+^ clusters (NeuN^+^/Iba-1^+^/Kv2.1^+^ cells) were significantly reduced in MCAO/R mice but substantially restored by Weisheng-tang (Figures 4A, B), despite Weisheng-tang reducing microglial coverage and P2Y12R coverage of neuronal cells (Figures 4C–E). These findings suggest that Weisheng-tang regulates communication between microglia and neurons by increasing neuronal Kv2.1 clusters while simultaneously suppressing the activation of microglial cells near neurons.

### 3.5 P2Y12R antagonist reversed the protective effects of Weisheng-tang

**FIGURE 5:**
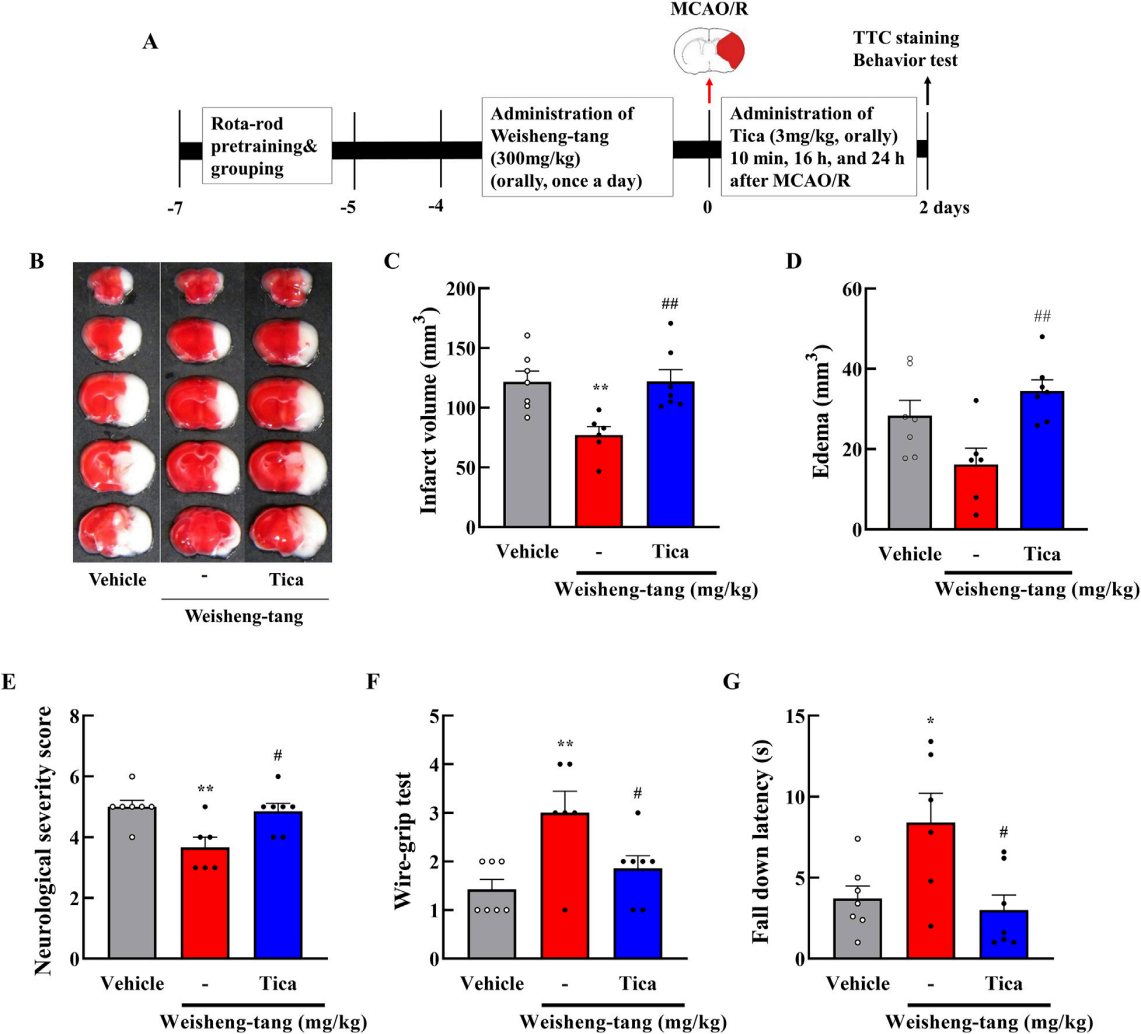
Ticagrelor reversed the protective effects of Weisheng-tang on ischemic stroke. **(A)** Experimental timeline of MCAO/R, Weisheng-tang/ticagrelor treatment, and behavioral tests. Mice were orally administered 300 mg/kg of Weisheng-tang for 4 days and 1 h before MCAO/R and ticagrelor (P2Y12R inhibitor, 3 mg/kg dissolved in 1% carboxymethyl cellulose) 10 min, 16 h, and 24 h after MCAO/R. **(B)** Representative images of 2% TTC-stained brain 48 h after MCAO/R. Quantification of **(C)** infarct volume and **(D)** edema using 2% TTC-stained brain (N = 6–7 each). **(E)** Neurological severity Scores, **(F)** wire-grip test, and **(G)** rotarod test assessed functional outcomes (N = 6–7 each). All data are expressed as means ± SEM. Statistical significance was assessed using one-way ANOVA with the Tukey’s *post hoc* test. *p < 0.05, **p < 0.01 vs. the vehicle group, #*p* < 0.05, ##*p* < 0.01 vs. 300 mg/kg Weisheng-tang group. Abbreviations: Ticagrelor (Tica), 2,3,5-triphenyltetrazolium chloride (TTC), and middle cerebral artery occlusion and reperfusion (MCAO/R).

Weisheng-tang appeared to increase P2Y12R expression, attenuating microglial activation and ultimately ameliorating post-stroke brain damage. To further validate this hypothesis, we investigated whether ticagrelor could reverse Weisheng-tang’s protective effects following ischemic brain injury. Figure 5 illustrates oral ticagrelor (3 mg/kg) administration alongside Weisheng-tang (300 mg/kg), which reversed Weisheng-tang’s protective effect on infarct and edema volume and neurological and motor functions. These results suggest that the tissue and functional recovery induced by Weisheng-tang following ischemic damage is mediated by P2Y12R.

## 4 Discussion

In this study, we demonstrated that Weisheng-tang protects against ischemic brain injury in MCAO/R mice, significantly reducing infarct volume and improving neurological and motor outcomes. Additionally, Weisheng-tang modulates microglial responses by reducing phagocytic microglia count, inducing morphological changes, and increasing P2Y12R expression on the microglia. Weisheng-tang enhances neuronal Kv2.1 clusters, thus contributing to improved microglia–neuron interactions. Notably, the P2Y12R antagonist ticagrelor reversed Weisheng-tang’s protective effects, underscoring P2Y12R’s critical role in mediating these beneficial outcomes in response to ischemic brain injury.

In a previous study, we demonstrated that Weisheng-tang significantly reduced ischemic damage 24 h after permanent ischemic injury in a mouse model of photothrombotic cortical ischemia (Kim et al., 2019). We investigated Weisheng-tang effects on neurobehavior assessments and infarct volume 48 h following MCAO/R. We observed a dose-dependent reduction in infarct volume, accompanied by improved neurological outcomes and motor function. These results suggest that pretreatment with Weisheng-tang can enhance tissue and functional recovery following MCAO/R. The results of this study corroborate with previous research on the neuroprotective properties of traditional botanical drugs in various cerebral ischemia models. These findings provide valuable insights into the potential therapeutic applications of Weisheng-tang in managing cerebral ischemic injury. However, we did not observe any long-term therapeutic effects of Weisheng-tang in neurological and motor function tests when using the same drug administration protocol as in the acute effect experiment—once daily for 4 days and 1 h before MCAO/R. Therefore, further studies with modifications in dosing amount, intervals, and duration of drug administration may be necessary to achieve sustained protective effects, as the current regimen does not appear to provide significant long-term protection.

In our initial assessment, we investigated whether Weisheng-tang influenced microglia. These cells, which are pivotal immune components within the central nervous system, play a crucial role in the brain’s response to injury and inflammation (Wang et al., 2007; Ceulemans et al., 2010). Postischemic microglial proliferation peaks at 48–72 h after focal cerebral ischemia and lasts for several weeks following the initial injury (Denes et al., 2007; Lalancette-Hébert et al., 2007). A significant finding of our study was the observed reduction in phagocytic microglia, identified by the CD68^+^/Iba-1^+^ phenotype (Hopperton et al., 2018), in the Weisheng-tang-treated group compared to the MCAO/R group. This finding suggests that Weisheng-tang significantly suppresses the activation of microglia, particularly those involved in phagocytosis. This reduction in phagocytic microglia activity may reflect the overall dampening of the inflammatory response in the ischemic brain, a promising outcome of stroke therapy (Fu et al., 2014). Despite the absence of a significant difference in Iba-1 expression, we observed notable morphological changes in the microglia among the three groups. The morphological transformation of microglia from a highly ramified resting state to an amoeboid-activated state in response to ischemic injury is well documented (Patel et al., 2013; Ma et al., 2017). In our study, MCAO/R-induced microglia activation, characterized by a reduction in the number of processes and an amoeboid appearance, was significantly reversed in the Weisheng-tang group. This reversal of microglial morphology indicated that Weisheng-tang facilitated the restoration of activated microglia, which are considered less pro-inflammatory and neurotoxic.

Furthermore, we observed a pronounced increase in P2Y12R expression on microglia following the Weisheng-tang treatment. P2Y12R is a well-established regulator of microglial process extension and retraction (Haynes et al., 2006). Microglial processes extend toward the ATP released from injured neuron cells through the metabotropic ATP receptor P2Y12R (Ohsawa et al., 2010). Another previous study suggested P2Y12R as a potential marker for anti-inflammatory microglial activation in ischemic rodents and human brains (Villa et al., 2018). Consistent with previous reports, P2Y12R was primarily localized within the microglia, as evidenced by its co-localization with Iba-1, a microglia-specific marker. Notably, P2Y12R^+^ and P2Y12R^+^/Iba-1^+^ cell counts were decreased in the peri-infarct area after MCAO/R, which was significantly reversed in the Weisheng-tang group. These findings suggest that Weisheng-tang promotes the restoration of retracted microglial processes in concert with P2Y12R expression, a crucial regulator of microglial process extension. The role of P2Y12R in mediating the protective effects of Weisheng-tang was further supported by a subsequent study using ticagrelor, a P2Y12R antagonist. Ticagrelor and clopidogrel have been used to treat cardiac and cerebrovascular diseases by inhibiting platelet P2Y12R expression to reduce thrombosis risk (Foster et al., 2001; Hollopeter et al., 2001). Administering ticagrelor with Weisheng-tang reversed the beneficial effects of Weisheng-tang, suggesting that tissue and functional recovery facilitated by Weisheng-tang following ischemic injury was, at least in part, mediated through the action of P2Y12R.

We investigated the impact of Weisheng-tang on microglia–neuron interactions concerning neuronal function following ischemic injury. Increased neuronal activation leads to microglial process coverage of the neuronal cell body, which depends on P2Y12R expression (Cserép et al., 2020). Microglia respond rapidly to changes in neuronal activity in the boundary zone of the infarct post-stroke (Szalay et al., 2016). We observed a significant reduction in Kv2.1^+^ clusters in MCAO/R mice, consistent with recent reports that Kv2.1 clustering at the microglial process and neuronal somatic membrane connection sites is reduced in the ischemic brain (Cserép et al., 2020). Kv2.1 channels are crucial for regulating neuronal excitability and are pivotal in neuronal function (Misonou et al., 2005). Additionally, clustered Kv2.1 proteins in neurons are crucial in creating exocytotic surfaces by anchoring vesicle fusion molecules to the neuronal membrane (Deutsch et al., 2012). The recovery of Kv2.1^+^ clusters by Weisheng-tang treatment suggests the potential restoration of normal neuronal function in the ischemic area, possibly contributing to improving post-stroke outcomes. Despite the increase in the number of P2Y12R^+^ and P2Y12R^+^/Iba-1^+^ cells by Weisheng-tang, we observed downregulated Iba-1 and P2Y12R expression around the neurons. This finding suggests that Weisheng-tang enhances neuronal function through microglia–neuron interaction by restoring Kv2.1 clustering while protecting neurons by decreasing the phagocytotic microglia covering them. These findings suggest that Weisheng-tang possesses a multifaceted mechanism of action for regulating communication between microglia and neurons. It enhances neuronal Kv2.1^+^ clusters while simultaneously suppressing phagocytic microglia near neurons. The regulation of microglia–neuron interactions and restoration of Kv2.1^+^ clusters suggests that Weisheng-tang’s neuroprotective effects extend beyond microglial modulation, impacting neuronal health and function.

The study findings, while promising, have some limitations. The use of very young mice might not fully represent the effects of Weisheng-tang in older or more varied populations, which are more commonly affected by stroke. The types of behavioral tests used in the experiment were limited, resulting in insufficient analysis of Weisheng-tang’s effects on various sensory and reflex neurobehavioral functions. Additionally, this study emphasizes the role of P2Y12R in mediating the effects of Weisheng-tang; however, it may oversimplify the complex mechanisms involved in ischemic brain injury, potentially neglecting other contributing factors, and signaling pathways. Moreover, this study did not explore the specific metabolites within Weisheng-tang responsible for the observed effects, which could be essential for developing more targeted therapies. This study offers promising insights into the potential therapeutic effects of Weisheng-tang on ischemic brain injury. Nonetheless, further research must address these limitations and validate these findings in clinical settings.

## 5 Conclusion

**FIGURE 6:**
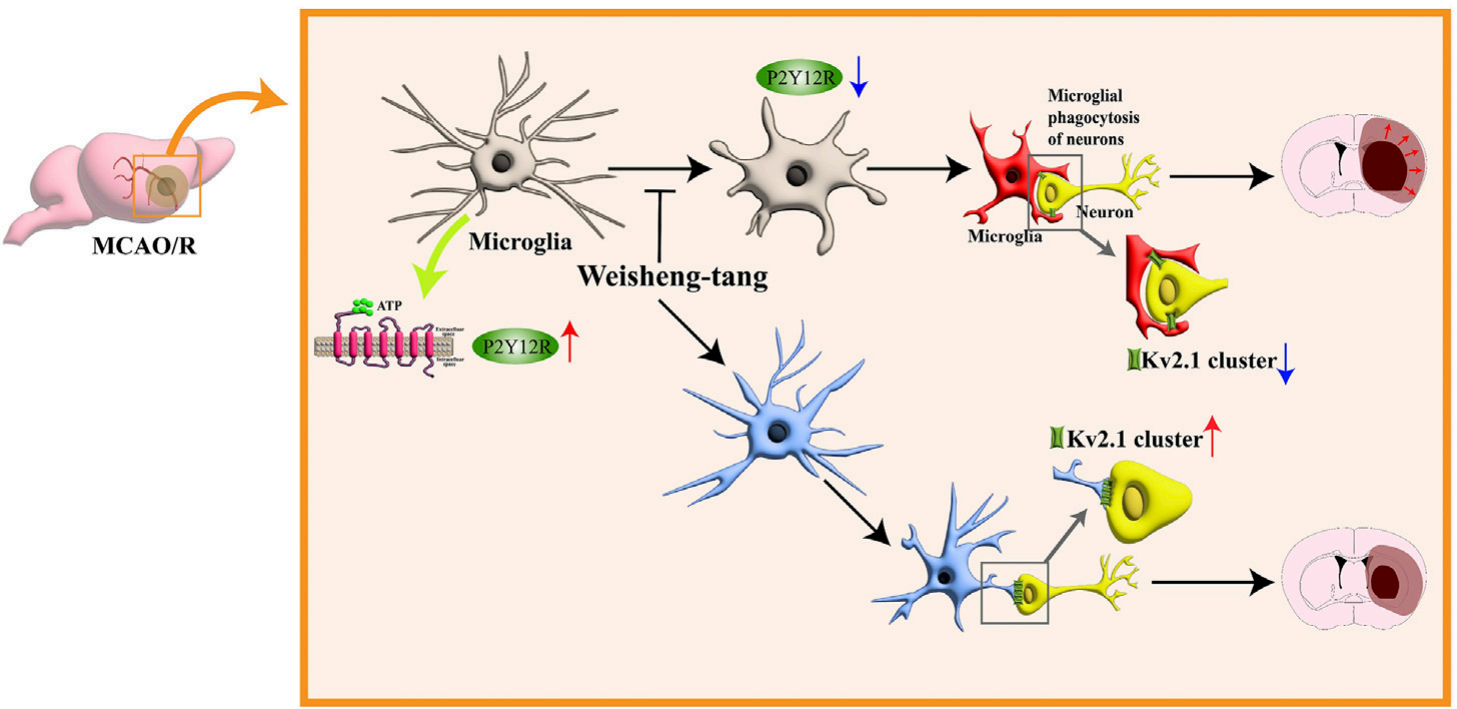
Schematic illustration for the protective effects of Weisheng-tang following MCAO/R. Weisheng-tang prevents microglial process retraction and neuronal phagocytosis by upregulating P2Y12R expression after MCAO/R. The regulation of microglia–neuron interactions and the restoration of Kv2.1^+^ clusters contribute to tissue and functional recovery, highlighting the neuroprotective potential of Weisheng-tang. Abbreviations: Adenosine triphosphate (ATP), voltage-gated potassium (Kv), and P2Y12 receptor (P2Y12R).

In conclusion, our study highlights the efficacy of Weisheng-tang in protecting against ischemic brain injury in MCAO/R mice by reducing infarct volume, improving neurological and motor outcomes, modulating microglial responses, and enhancing microglia–neuron interactions. These findings suggest the potential therapeutic value of Weisheng-tang in managing ischemic stroke (Figure 6).

## Data Availability

The raw data supporting the conclusions of this article will be made available by the authors, without undue reservation.
