# Supplementary material for: Weisheng-tang protects against ischemic brain injury by modulating microglia activation through the P2Y12 receptor
Source: Front Pharmacol. 2024 Sep 4;15:1347622. doi: 10.3389/fphar.2024.1347622 (PMC11408171; doi:10.3389/fphar.2024.1347622)
Supplement: Supplementary file 1 [file Presentation1.PPTX]

## Slide 1
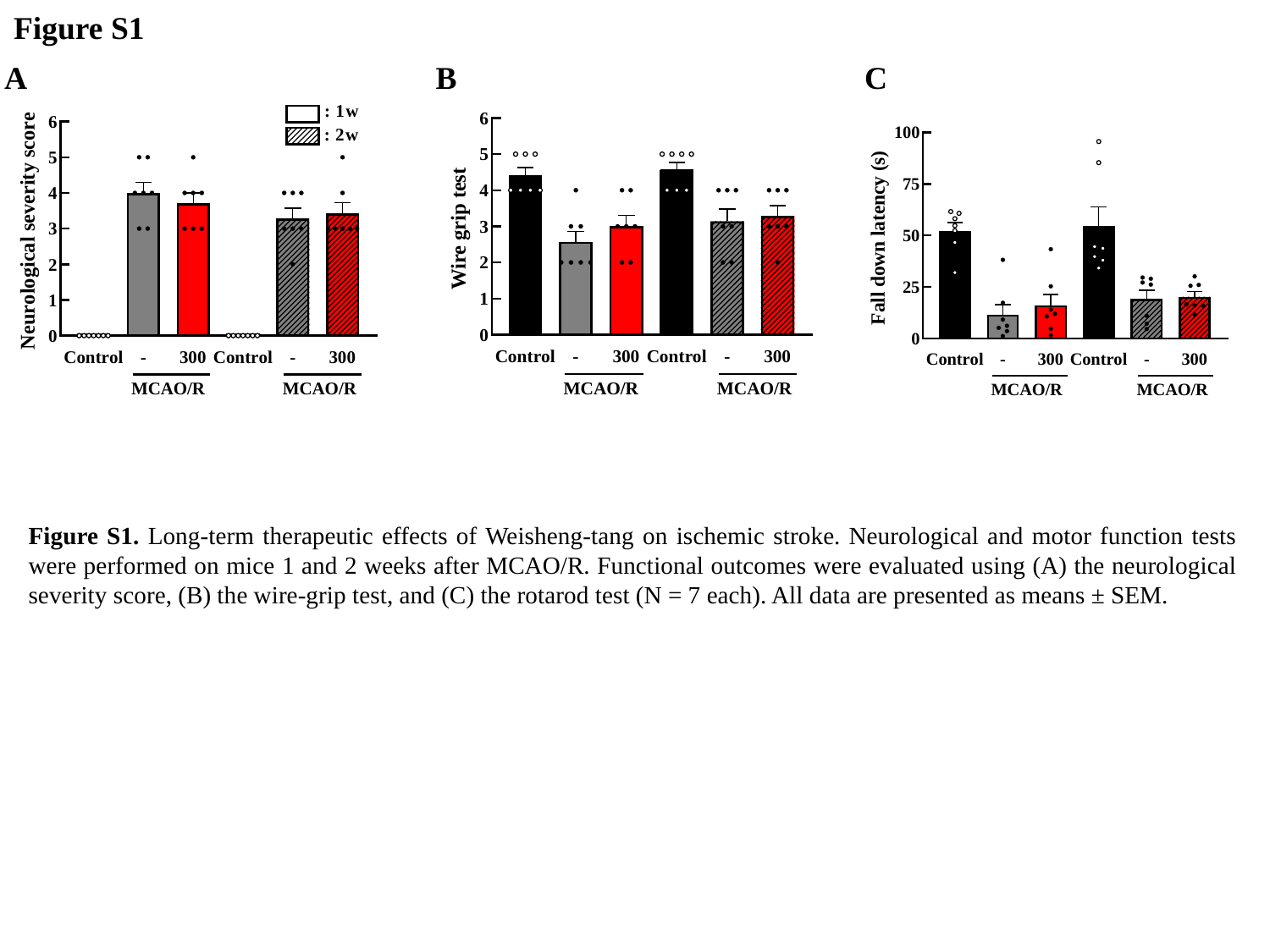

Figure S1
A
B
C
Figure S1. Long-term therapeutic effects of Weisheng-tang on ischemic stroke. Neurological and motor function tests were performed on mice 1 and 2 weeks after MCAO/R. Functional outcomes were evaluated using (A) the neurological severity score, (B) the wire-grip test, and (C) the rotarod test (N = 7 each). All data are presented as means ± SEM.

## Slide 2
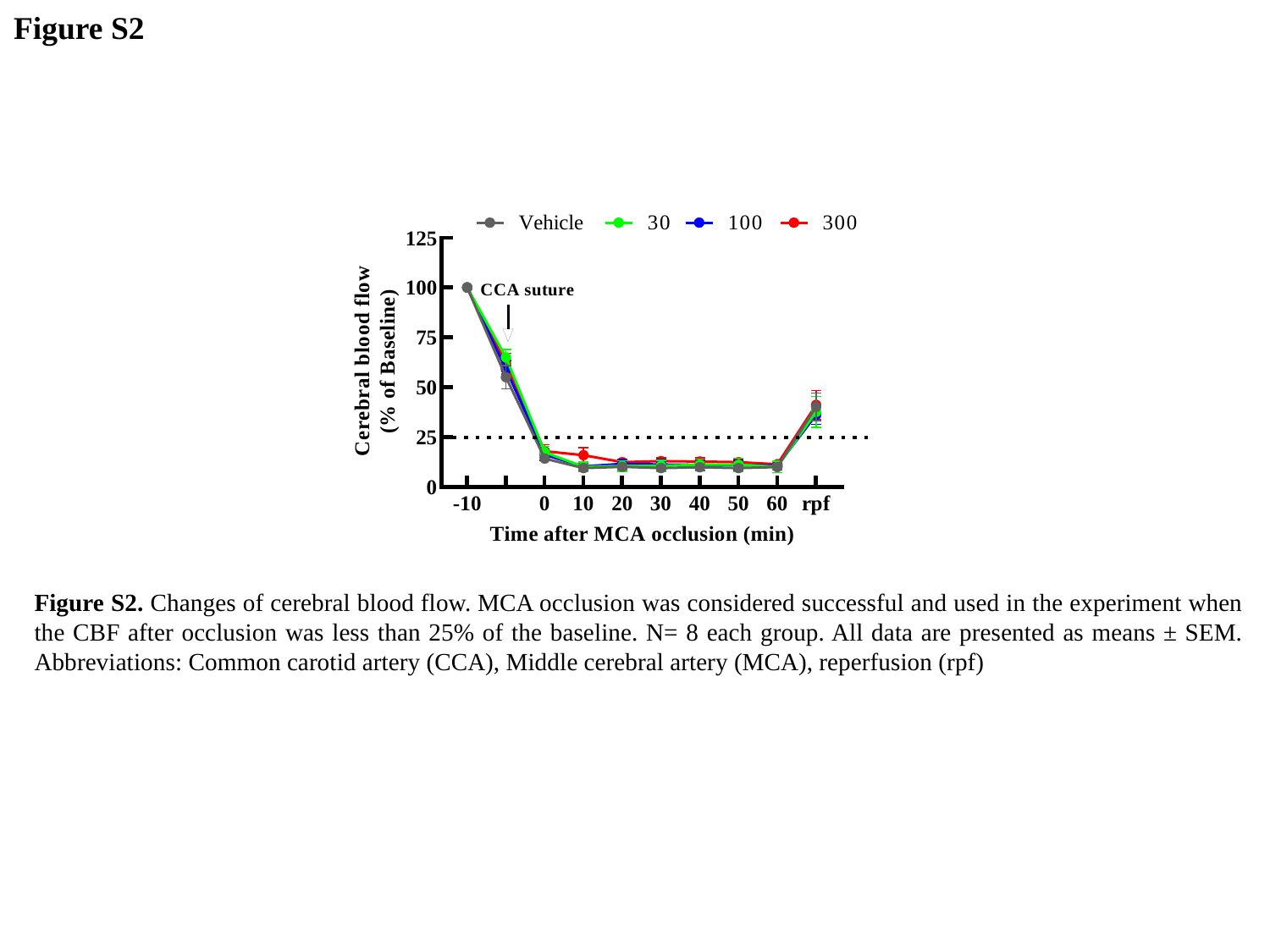

Figure S2
Figure S2. Changes of cerebral blood flow. MCA occlusion was considered successful and used in the experiment when the CBF after occlusion was less than 25% of the baseline. N= 8 each group. All data are presented as means ± SEM. Abbreviations: Common carotid artery (CCA), Middle cerebral artery (MCA), reperfusion (rpf)

## Slide 3
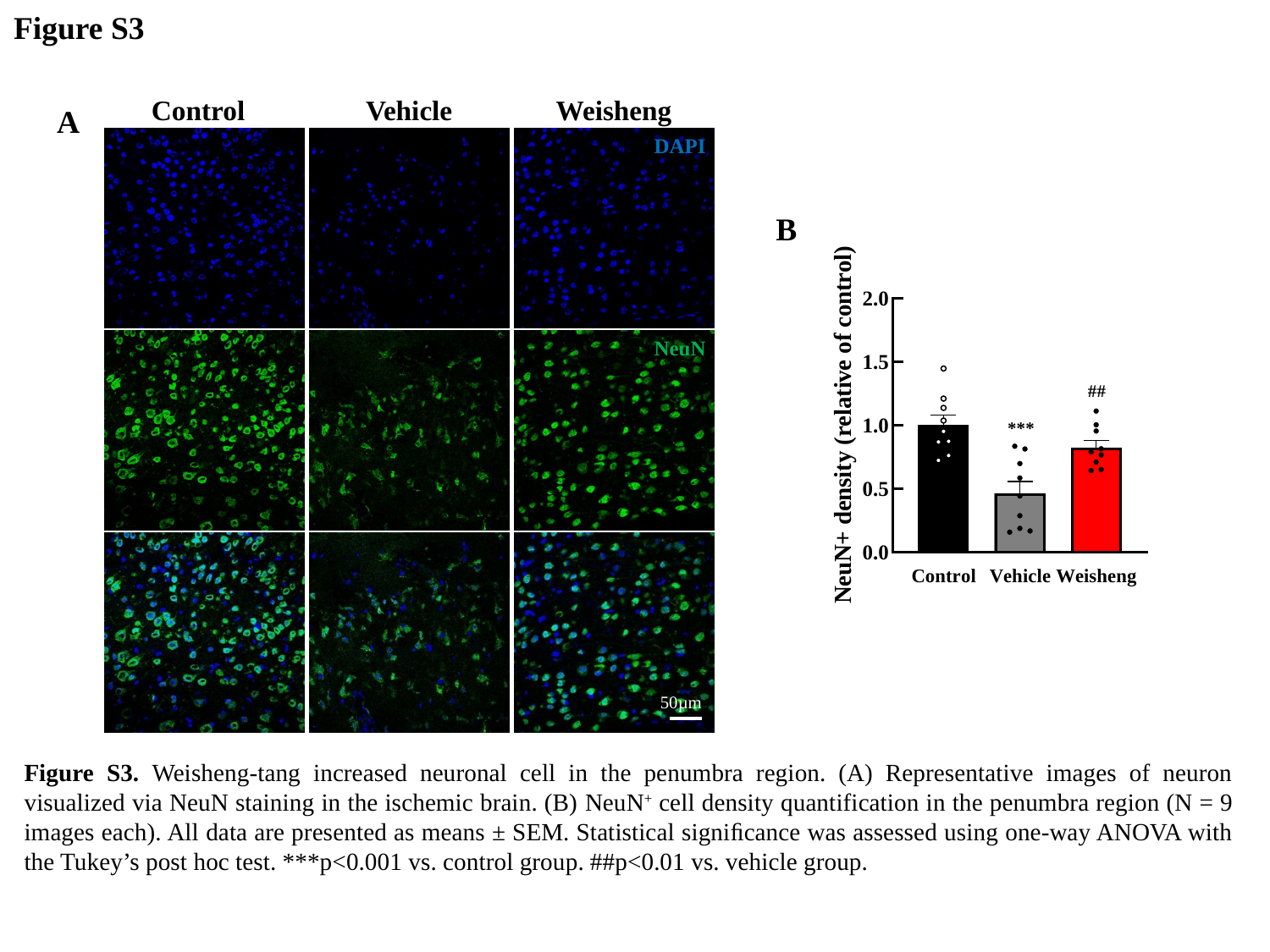

Figure S3
Weisheng
Vehicle
Control
A
DAPI
B
NeuN
50µm
Figure S3. Weisheng-tang increased neuronal cell in the penumbra region. (A) Representative images of neuron visualized via NeuN staining in the ischemic brain. (B) NeuN+ cell density quantification in the penumbra region (N = 9 images each). All data are presented as means ± SEM. Statistical signiﬁcance was assessed using one-way ANOVA with the Tukey’s post hoc test. ***p<0.001 vs. control group. ##p<0.01 vs. vehicle group.
